# Effect of early supported discharge after stroke on patient reported outcome based on the Swedish Riksstroke registry

**DOI:** 10.1186/s12883-019-1268-8

**Published:** 2019-03-12

**Authors:** Anna Bråndal, Marie Eriksson, Eva-Lotta Glader, Per Wester

**Affiliations:** 1 Department of Public Health and Clinical Medicine, Umeå University, S-901 87 Umeå, Sweden; 2 Department of Community Medicine and Rehabilitation, Physiotherapy, Umeå University, S-901 87 Umeå, Sweden; 3 Department of Statistics, Umeå School of Business and Economics Umeå University, S-901 87 Umeå, Sweden; 4 Department of Clinical Sciences, Karolinska Institute, Danderyd hospital, S-182 88 Stockholm, Sweden

**Keywords:** Stroke, Early supported discharge, Rehabilitation, Patient reported outcome measurement

## Abstract

**Background:**

The efficacy of early supported discharge (ESD) has not been tested in current stroke care setting, which provide relatively short hospital stays, access to hyper-acute therapies and early carotid stenosis interventions. This study aimed to compare patient-reported outcome measures (PROM) among patients with stroke that received modern stroke unit care with or without ESD.

**Methods:**

Observational study of 30,232 patients with first-ever stroke registered in the Riksstroke registry in Sweden, between 1 January 2010 and 31 December 2013. Patient characteristics were collected from the Riksstroke and Statistics Sweden databases. The primary outcome was satisfaction with the rehabilitation at 3 months after discharge. Secondary outcome were information about stroke provided, tiredness/fatigue, pain, dysthymia/depression, general health status and dependence in activities of daily living (mobility, toileting and dressing) at 3 months after the stroke. We used separate multivariable logistic regression models for each PROM variable to analyze associations between PROMs and ESD/no ESD.

**Results:**

The ESD group comprised 1495 participants: the control group comprised 28,737 participants. Multivariable logistic regression models of PROMs showed that, compared to controls, the ESD group was more satisfied with rehabilitation after discharge (OR: 1.78, 95% CI: 1.17–2.49), experienced less dysthymia/depression (OR: 0.68, 95% 0.55–0.84) and showed more independence in mobility (OR: 1.50, 95% CI: 1.17–1.92), toileting (OR: 1.30, 95%CI: 1.05–1.61), and dressing (OR: 1.23, 95%CI: 1.02–1.48).

**Conclusion:**

In the setting of modern stroke unit care, ESD appeared to have positive effects on stroke rehabilitation, in the subacute phase.

## Background

Early supported discharge (ESD) with continued rehabilitation in the home has been shown to be beneficial among patients with mild to moderate stroke. The ESD- model for rehabilitation was introduced in the late 1990s and includes an interdisciplinary team with appropriate recourses that coordinates the discharge and plan, supervise and continue the rehabilitation in the home environment [1]. This form of rehabilitation accelerates the discharge from hospital, reduce long term dependency and admission to institutional care [1–4]. However, the criticism has been raised that most of the randomized controlled trials on ESD services were published more than 10 years ago [5, 6]. Today, the majority of patients are being discharged home early after stroke, due to access of hyper-acute therapies, and implement early interventions for carotid stenosis. There is a need to evaluate efficacy and safety of ESD in current stroke care settings and to adapt ESD service to local conditions for appropriate implementation [1, 5, 6].

In Sweden, the majority (91% in 2014) of patients with stroke are cared for at a stroke unit (http://www.riks-stroke.org), but there has been no major expansion of ESD. Despite recommendations in the National Guidelines for stroke care [7], the proportion of patients with stroke that receive ESD after stroke unit care has varied, dramatically across Sweden. Västerbotten County (Umeå Stroke Center and Skellefteå hospital) is one of the counties with high a proportion of ESDs (http://www.riks-stroke.org, [8]).

We previously described the method, content, and outcome of ESDs according to the Umeå Stroke Center model in Västerbotten County [9]. This model showed that it was possible to adopt and implement ESD for patients with stroke in Umeå. The model included important key elements for an effective ESD service [6, 8], such as a multidisciplinary team with experience in stroke rehabilitation, appropriate resources, periodic team meetings, and continuous evaluations of outcome with standardized measurements. Our previous results showed that ESD services reduced patient dependence in activities in daily living (ADL) and increased patient mobility, without increasing the risk of accidental falls or other injuries [9]. The patients were very satisfied with the ESD-service. However, that observational implementation study did not include a control group.

The present study aimed to evaluate patient-reported outcomes measures (PROMs) among patients with stroke that received modern stroke unit care, and compare PROMs between those that received or did not receive ESD. The ESD was delivered according to a previously described model [9]. We hypothesized that patients that received ESD would exhibit improved PROMs regarding satisfaction with rehabilitation (primary outcome), activity in daily living (ADL), tiredness/fatigue, pain, dysthymia/depression, general health status and information about stroke (secondary outcomes) compared to controls.

## Methods

For this case control, observational study, we retrieved data from the Swedish Stroke Registry, Riksstroke [10], and from the Longitudinal Integration Database for Health Insurance and Labor Market Studies (LISA). Information from the Swedish Stroke Registry was linked to the LISA database through personal identification numbers. This study was approved by the Regional Ethics Review Board at Umeå University (Dnr 2012–179-32 M, 2014–273-32 M).

### Register

Currently, all 72 hospitals that treat patients with acute stroke participate in the Swedish Stroke Registry, Riksstroke [10], which started in 1994. The primary aim of Riksstroke is to monitor and support improvements in the quality and implementation of new methods in stroke care in Sweden. The registry includes patients with ischemic and hemorrhagic stroke and data on first-ever and recurrent strokes. The acute phase questionnaire of Riksstroke contains basic patient characteristics (age, sex, living conditions, history of previous stroke, and comorbidities), diagnosis, level of consciousness on arrival, pharmaceutical treatments, complications, and the sequence of care (type of stroke care, organization, and department). In Riksstroke the hospitals that care for patients with acute stroke have been divided into three categories: university hospitals (9 pcs), specialized nonuniversity hospitals (23 pcs) and community hospitals (40 pcs) [11]. Riksstroke also includes 3-month and 12-month follow up questionnaire that describe patient-reported outcomes and rehabilitation after stroke. The 3-month and 12-month questionnaire is administrated by the hospitals and filled in by the patients.

The LISA database at Statistics Sweden includes information on all Swedish citizens, starting at 16 years of age. In particular, it includes socioeconomic factors, like disposable income, education, and country of birth.

### Participants and setting

**Fig. 1:**
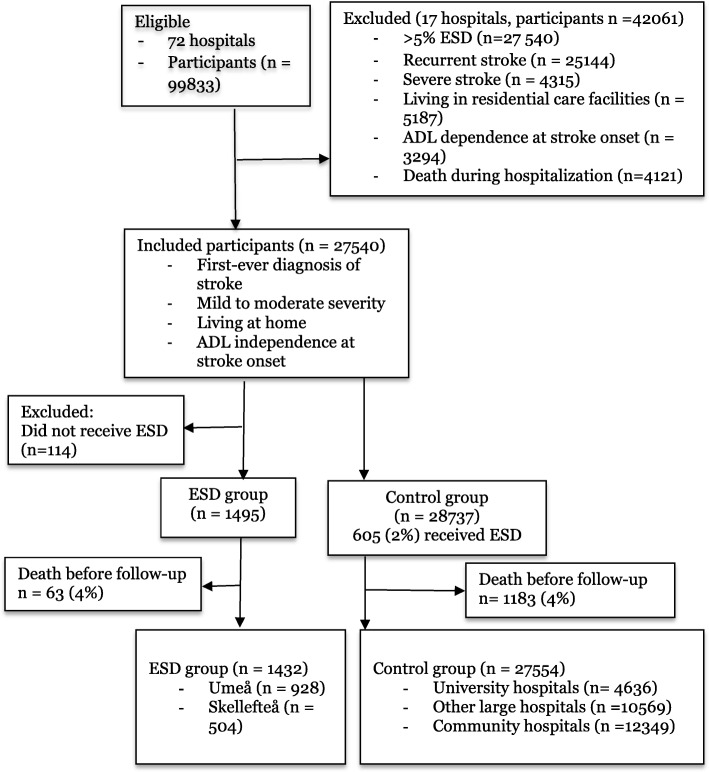
Flow chart of the study inclusion procedure. ESD: early supported discharge

All patients registered in the Riksstroke registry with a first-ever diagnosis of acute stroke between 1 January, 2010 and 31 December 2013 were included in the present study, when they fulfilled the following criteria: diagnosis of ischemic or hemorrhagic stroke; mild to moderate stroke severity at admission (measured as level of consciousness on a scale of 1–3, according to the Reactive Level Scale, RLS85) [12]; living at home; and independency in ADL at stroke onset. Patients that met the inclusion criteria were divided into an ESD intervention group and a control group (Fig. 1).

### ESD intervention group

Västerbotten County have three hospitals with different primary catchment areas that cared patients in acute and sub-acute phases of stroke. Two of these hospitals, the Umeå Stroke Center (university hospital) and Skellefteå hospital (community hospital) had similar organizations regarding stroke care. Both hospitals had stroke unit care followed by ESD according to a previously described model [9]. These hospitals provided ESD to a comparatively large proportion of patients (41.2% at Umeå Stroke Center and 57.5% at Skellefteå hospital) compared to other hospitals in Sweden. The intervention group (ESD group) consisted of consecutive patients with stroke that received modern stroke unit care, followed by the ESD-service. During the study period, ESD was delivered to 41.2% of all patients with stroke admitted to the Umeå Stroke Center and 57.5% admitted to Skellefteå. Patients with severe stroke and those who died during hospitalization were not included in the ESD intervention group.

### Control group

The control group consisted of patients treated at stroke units in hospitals that cared for patients with acute stroke. At these hospitals, a low (< 5% of all stroke patients) proportion of patients were given ESD. Therefore, the control group, which fulfilled the same inclusion criteria as the intervention group also included some patients that received ESD (< 5%). Patients with severe stroke and those who died during hospitalization were not included in the control group. Hospitals in Sweden that only partially implemented ESD (5–20% of stroke patients) and those who registered other models of home rehabilitation were excluded.

### Variables

Patient characteristics for both groups included sex, age, stroke subtype, treatment with thrombolysis, domestic companions, mobility, hypertensive treatment, diabetes, atrial fibrillation, and smoking before stroke onset. The level of consciousness upon admission and the length of hospital stay were also considered baseline characteristics of the two groups. These variables were retrieved from the Riksstroke registry. Information on education and country of birth was retrieved from the LISA database. The level of education was classified as primary school, secondary school, or university level. Country of birth was categorized as Sweden, Nordic countries (Sweden excluded), Europe (Nordic countries excluded), or other countries.

The outcome variables for this study were PROM results from the 3-months follow up recorded in Riksstroke. The primary outcome was satisfaction with the rehabilitation after discharge. Secondary outcome variables were; satisfaction with information provided about stroke, tiredness/fatigue, pain, dysthymia/depression, general health status and ADL dependence (mobility, toileting, and dressing). Data from the PROM values recorded in Riksstroke at the 3-months follow up have been validated against established measurements with the finding of accurate reliability (http://www.riks-stroke.org).

Some questions had multiple choice responses, and for our analysis, the responses were dichotomized. For example, patients were asked the questions: “How satisfied or dissatisfied are you with the rehabilitation or training you received after your stay in the hospital?” and “How satisfied or dissatisfied are you with the stroke information provided?”; and they responded with one of the following options: very satisfied, satisfied, dissatisfied, very dissatisfied, no need of rehabilitation, needed, but did not receive rehabilitation, or I do not know. And dependent categories were taken as reference (code = ref) for variables measuring a favourable outcome (satisfaction with rehabilitation, information about stroke, general health and Adl function) When assessing outcome variables that means an unfavourable result (tiredness/fatigue, pain, depression) we used a ‘positive’ reference (code = ref).

Similarly, the questions: “Do you feel tired/fatigue?”, “Do you have any pain?”, and “Do you feel depressed?” had response options; never, almost never, sometimes, often, constantly, or do not know, and they were coded as often or seldom (ref). The “often” code was assigned to: sometimes, often, constantly, and missing. The question “How would you assess your general health?” had response options: very good, somewhat good, somewhat poor, very poor, and they were coded good or poor(ref). The “poor” code was assigned to somewhat poor, very poor, and do not know.

Finally, responses to the ADL questions were coded as independent and dependent (ref), as follows. The question: “How is your mobility now?” had response options: “I can get around by myself indoors and outdoors” (independent), “I can get around by myself indoors, but not outdoors” (independent), “I need assistance when I want to move around” (dependent), or “I do not know” (dependent). The question: “Do you need assistance when visiting the toilet?” had response options: “I can manage visiting the toilet by myself” (independent) and “I need assistance when visiting the toilet” (dependent). The question “Do you need assistance getting dressed and undressed?” had response option: “I can manage to get dressed and undressed by myself”, (independent) and “I need assistance getting dressed and undressed” (dependent).

### Statistical analysis

Baseline characteristics for the ESD and control groups are expressed as frequencies and proportions, for categorical data, and as the mean and standard deviation (SD), for continuous variables. Baseline values were compared between groups with the independent t-test (for continuous variables) and the chi-square test (for categorical variables). The association between PROMs and ESD were analysed in separate multivariable logistic regression models. These models assessed the probability of satisfaction with rehabilitation after discharge, satisfaction with the information provided about stroke, tiredness/fatigue, pain, dysthymia/depression, general health status, and ADL dependency (mobility, toilet hygiene, and dressing) in the ESD group compared to the control group. In addition to the group effect (ESD or control), each model included the independent factors that were significant in the baseline comparison: age (continuous), thrombolysis, smoking, atrial fibrillation, country of birth, and education. The results are presented as the odds ratio (OR) with a 95% confidence interval (95% CI). The confidence intervals and *P*-values were not adjusted for multiple testing. Sensitivity analyses were performed where the missing data (missing or unknown) were included in the reference category. Subgroup analyses were performed to compare data for Umeå Stroke Center with other university hospitals and Skellefteå hospital with community hospitals. In the subgroup analysis, missing data were included in the reference category. The analyses were performed with IBM SPSS Statistics 21 statistical software.

## Results

Between 1 January 2010 and 31 December 2013, 99,833 patients whereof 74,689 with a first-ever diagnosis of acute stroke were registered in the Riksstroke. Of these patients, 30,232 were included in this study (Fig. 1). During these years, the coverage rate in The Riksstroke varies from 88 to 91%. The ESD group comprised of 1495 participants that received ESD treatment after stroke unit care at the Umeå Stroke Center (university hospital) and at Skellefteå hospital (community hospital). The control group comprised 28,737 participants treated at 3 university hospitals, 13 other large hospitals, and 39 community hospitals. The control group were treated at stroke units where a very low proportion (605 participants, 2%) received ESD. Participants in the ESD group were slightly younger, had a higher education level and were more often born in Sweden than participants in the control group. The ESD group had lower frequencies of smoking and atrial fibrillation and a higher frequency of thrombolysis than the control group (Table 1).

**Table 1:** Baseline characteristics of study participants (*n* = 30,232)

Variable	ESD group	Control group	*p* value
	(*n* = 1495)	(*n* = 28,737)	
Sex, n (%)			0.212
Males	829 (55.5)	15,461 (53.8)	
Females	666 (44.5)	13,276 (46.2)	
Age, mean (SD)	73 (12.8)	74 (12.4)	0.001
Stroke subtype, n (%)			0.244
Ischemic Stroke	1314 (87.9)	25,646 (89.2)	
Intracerebral hemorrhage	159 (10.6)	2741 (9.5)	
Undetermined	22 (1.5)	350 (1.2)	
Thrombolysis, n (%)	212 (14.2)	2252 (7.8)	0.001
Missing	1 (0.1)	142(0.5)	
Living alone, n (%)	605 (40.5)	12,428 (43.2)	0.091
Missing data	4 (0.3)	97 (0.3)	
Mobility, n (%)			0.249
Independent indoors- and outdoors	1468 (98.2)	28,088 (97.7)	
Independent indoors	27 (1.8)	649 (2.3)	
Hypertension, n (%)	817 (54.6)	16,430 (57.2)	0.076
Missing data	3 (0.2)	107 (0.4)	
Diabetes, n (%)	250 (17.4)	5254 (18.3)	0.106
Missing data	0 (0.0)	40 (0.1)	
Atrial fibrillation, n (%)	282 (18.9)	6847 (23.8)	0.001
Missing data	0 (0.0)	102(0.4)	
Smoking, n (%)	157 (10.5)	4331(15.1)	0.001
Missing data	36(2.4)	2001 (7.0)	
Level of consciousness on admission, n (%)			0.123
RLS 1	1391 (93.0)	26,419 (91.9)	
RLS 2–3	104 (7.0)	2318 (8.1)	
Length of hospital stay (SU), median (Q1-Q2)	5 (3–10)	6 (3–13)	0.920
Missing data	50 (3.3)	1220 (4.2)	
Education, n (%)			0.001
Primary School	527(35.3)	11,902 (41.4)	
Secondary School	590 (39.5)	9930 (34.6)	
University	259 (17.3)	4428(15.4)	
Missing data	119 (7.9)	2470 (8.6)	
Country of birth, n (%)			0.001
Sweden	1431 (95.7)	25,392 (88.4)	
Nordic countries ^a^	41 (2.7)	1436 (5.0)	
Europe ^b^	11 (0.8)	1234 (4.3)	
Other countries	9 (0.6)	466 (1.6)	
Missing data	3 (0.2)	209 (0.7)	

SU - Stroke unit

^a^Except Sweden

^b^Except Nordic Countries

Three months after a stroke, 28,986 participants remained alive: 1432 (96%) were in the ESD group and 27,554 (96%) were in the control group. Due to differences between the ESD and control participants in the ESD groups at baseline the multivariable logistic regression models of PROMs were adjusted for age, thrombolysis, smoking, atrial fibrillation, country of birth, and education. The analysis showed that the ESD compared with the control group were more satisfied with rehabilitation after discharge (*p* < 0.004, OR 1.78, 95% CI: 1.17–2.49), showed more independence in mobility (*p* < 0.001, OR 1.50, 95% CI: 1.17–1.92), toileting (*p* < 0.016, OR 1.30, 95% CI: 1.05–1.61), and dressing (*p* < 0.03, OR 1.23, 95% CI: 1.02–1.48), and felt less dysthymia/depression (*p* < 0.001, OR 0.68, 95% CI: 0.55–0.84) (Table 2). There were no significant differences between the groups in the information received about stroke, tiredness/fatigue, pain or general health status (Table 2).

**Table 2:** Multiple logistic regression of patient reported outcome variables 3 months after stroke

(*n* = 28,986) Odds ratio (OR) with 95% confidence interval						
Variable	ESD group	Control group	*p* value	0R	95% CI	
	(*n* = 1432)	(*n* = 27,554)			Lower	Upper
Satisfaction with rehabilitation^a^, n (%)						
Satisfied	507 (35.4)	9182 (33.3)	0.001	1.78	1.17	2.49
Dissatisfied	47(3.3)	1405 (5.1)		ref		
No need	449 (31.4)	9231 (33.5)				
Not received	44 (3.1)	1501 (5.4)				
Missing and do not know	385 (26.9)	6235 (22.6)				
Information provided about stroke, n (%)						
Satisfied	927 (64.7)	16,820 (61.0)	0.533	1.08	0.85	1.37
Dissatisfied	82 (5.7)	1700 (6.2)		ref		
No need	87 (6.1)	3256 (11.8)				
Missing and do not know	336 (23.5)	5778 (21.0)				
Tiredness/fatigue, n (%)						
Seldom	780 (54.5)	15,440 (56.0)		ref		
Often	410 (28.6)	8595 (31.2)	0.748	0.98	0.86	1.11
Missing	242 (16.9)	3519 (12.8)				
Pain, n (%)						
Seldom	956 (66.8)	19,117 (69.4)		ref		
Often	217 (15.2)	4745 (17.2)	0.523	0.95	0.814	1.11
Missing	259 (18.1)	3692 (13.4)				
Depression, n (%)						
Seldom	1083 (75.6)	20,819 (75.6)		ref		
Often	109 (7.6)	3118 (13.3)	0.001	0.68	0.55	0.84
Missing	240 (16.8)	3617 (13.1)				
General health, n (%)						
Good	988 (69.0)	19,163 (69.5)	0.091	1.15	0.98	1.35
Poor	200 (14.0)	4779 (17.3)		ref		
Missing	244 (17.0)	3612 (13.1)				
Adl-independence, n (%)						
Mobility						
Independent	1146 (80.0)	21,722 (78.8)	0.001	1.50	1.17	1.92
Dependent	83 (5.8)	2564 (9.3)		ref		
Missing	203 (14.2)	3268 (11.9)				
Toileting						
Independent	1117 (78.0)	21,236 (77.1)	0.016	1.30	1.05	1.61
Dependent	112 (7.8)	3247 (11.8)		ref		
Missing	203 (14.2)	3247 (11.8)				
Dressing						
Independent	1075 (75.1)	20,394(74.0)	0.030	1.23	1.02	1.48
Dependent	155 (10.8)	3895 (14.1)		ref		
Missing	202 (14.1)	3265 (11.8)				

^a^ After Discharge

The proportions of missing responses were higher in the ESD group for all variables (14.1–26.9% missing responses) than in the control group (11.8–22.6% missing responses). In a sensitivity analysis, where missing/unknown/no need responses were included in the reference category, the ESD group remained more satisfied with rehabilitation after discharge (*p* < 0.001, OR 1.26, 95% CI: 1.10–1.43) and felt less depressed (*p* < 0.001, OR 0.68, 95% CI: 0.55–0.84) than controls, but the groups were not significantly different in the other variables (Table 3).

**Table 3:** Patient-reported outcome variables, 3 months after stroke (n = 28,986) (sensitivity analysis)

Variable	ESD group	Control group	*p* value	0R	95% CI	
	(*n* = 1432)	(*n* = 27,554)			Lower	Upper
Satisfaction with rehabilitation^a^, n (%)						
Satisfied	507 (35.4)	9182 (33.0)	0.001	1.26	1.10	1.43
Dissatisfied^b^	925 (64.6)	18,372 (67.0)		ref		
Information provided about stroke, n (%)						
Satisfied	927 (64.7)	16,820 (61.0)	0.444	1.05	0.93	1.17
Dissatisfied^b^	505 (35.3)	10,734 (39.0)		ref		
Tiredness/fatigue, n (%)						
Seldom	780 (54.5)	15,440 (56.0)		ref		
Often and missing	652 (45.5)	12,114 (44.0)	0.750	0.98	0.66	1.11
Pain, n (%)						
Seldom	956 (66.8)	19,117 (69.4)		ref		
Often and missing	476 (33.2)	8437 (30.6)	0.523	0.95	0.81	1.11
Depression, n (%)						
Seldom	1083 (75.6)	20,819 (75.6)		ref		
Often and missing	349 (24.4)	6735 (24.4)	0.001	0.68	0.55	0.84
General health, n (%)						
Good	988 (69.0)	19,163 (69.5)		ref		
Poor and missing	444 (31.0)	8391 (30.5)	0.090	0.87	0.74	1.02
Adl-independence, n (%)						
Mobility						
Independent	1109 (93.0)	21,722 (78.8)	0.001	0.78	0.68	0.90
Dependent and missing	323 (22.5)	5832 (21.2)		ref		
Toileting						
Independent	1132 (78.9)	21,236 (77.1)	0.001	0.78	0.68	0.88
Dependent and missing	352 (24.5)	6318 (22.9)		ref		
Dressing						
Independent	1077 (75.2)	20,394(74.0)	0.001	0.81	0.71	0.92
Dependent and missing	355 (24.8)	7169 (26.0)		ref		

^a^After Discharge

^b^Dissatisfied includes very dissatisfied, dissatisfied, in need, but did not received rehabilitation, I do not know and missing

In the subgroup analyses of data form the Umeå Stroke Center (ESD subgroup 1) and other university hospitals (control subgroup 1), we found that the ESD group 1 was more satisfied with the rehabilitation (*p* < 0.0001, OR2.27, 95% CI: 1.44–3.59) and stroke information provided (*p* < 0,001, OR 1.93, 95% CI: 1.32–2.37) and experienced less depression (*p* < 0.001, OR 0.55, 95% CI: 0.41–0.75), and were better in Adl function than the control subgroup 1 (Table 4). In the subgroup analysis of data from Skellefteå hospital (ESD subgroup 2) and other community hospitals (control subgroup 2), there were no significant differences. (Table 4).

**Table 4:** Subgroups analysis of patient-reported outcome variables 3 months after stroke (*n* = 18,417)

	University hospital				Small hospitals			
Variable	ESD 1 (*n* = 928)	Control 1 (*n* = 4636)	*p value*	0R (95% CI)	ESD 2 (*n* = 504)	Control 2 (*n* = 12,349)	*p value*	0R (95% CI)
Satisfaction with rehabilitation*, n (%)								
Satisfied	338 (36.4)	1658 (35.8)	0.001	2.27 (1.44–3.58)	169 (33.5)	4024 (32.6)	0.863	1.04 (0.66–1.64)
Dissatisfied	25 (2.7)	278 (6.0)		ref	22 (4.4)	593 (4.8)		ref
No need	267 (28.8)	1342 (28.9)			182 (36.1)	4401 (35.6)		
Not received	20 (2.2)	215 (4.6)			24 (4.8)	732 (5.9)		
Missing and do not know	278 (30.0)	1143 (24.7)			107 (21.2)	2599 (21)		
Information about stroke, n (%)								
Satisfied	590 (63.6)	2626 (56.6)	0.001	1.93 (1.32–2.73)	337 (66.9)	7683 (62.2)	0.06	0.63 (0.45–0.88)
Dissatisfied	38 (4.1)	337 (7.3)		ref	44(8.7)	676 (5.5)		ref
No need	52 (5.6)	595 (12.8)			35 (6.9)	1531 (12.4)		
Missing and do not know	248 (26.7)	1078 (23.3)			88 (17.5)	2459 (9, 19)		
Tiredness/fatigue, n (%)								
Seldom	470 (50.6)	2337 (50.4)		ref	310 (61.5)	7256 (58.8)		ref
Often	252 (27.2)	1553 (33.5)	0.117	0.87 (0.74–1.04)	150 (29.8)	3622 (29.3)	0.860	0.98 (0.80–1.20)
Missing	206 (22.2)	746 (16.1)			44 (8.7)	1471 (11.9)		
Pain, n (%)								
Seldom	586 (63.1)	3074 (66.3)		ref	370 (73.4)	8794 (71.2)		ref
Often	121 (13.0)	756 (16.3)	0.215	0.87 (0.70–1.08)	79 (15.7)	1942 (15.7)	0.976	1.04 (0.78–1.29)
Missing	221 (23.8)	806 (17.4)			55 (10.9)	1613 (13.1)		
Depression, n (%)								
Seldom	660 (71.1)	3323 (71.7)		ref	423 (83.9)	9586 (77.6)		ref
Often	52 (5.6)	507 (10.9)	0.001	0.55 (0.41–0.75)	35 (6.9)	1104 (8.9)	0.09	0.73 (0.51–1.05)
Missing	216 (23,3)	806 (17.4)			46 (9.1)	1659 (13.4)		
General health, n (%)								
Good	604 (65.1)	3012 (65.0)	0.081	1.23 (0.98–1.54)	384 (76.2)	8847 (71.6)	0.57	1.08 (0.82–1.42)
Poor	110 (11.9)	743 (16.0)		ref	67 (13.3)	1705 (13.8)		ref
Missing	214 (23.1)	881 (19.0)			53 (10.5)	1797 (14.6)		
Adl-independence, n (%)								
Mobility								
Independent	683 (73.6)	3503 (75.6)	0.001	1.97 (1.38–2.80)	426 (84.5)	9868 (79.9)	0.106	1.34 (0.94–1.90)
Dependent	45 (4.8)	428 (9.2)		ref	38 (7.5)	1214 (9.8)		ref
Missing	200 (21.6)	705 (15.2)			40 (7.9)	1267 (10.3)		
Toileting								
Independent	670 (72.2)	3451 (74.4)	0.001	1.68 (1.23–2.30)	410 (81.3)	9609 (77.8)	0.533	1.10 (0.81–1.49)
Dependent	58 (6.3)	484 (10.4)		ref	54 (10.7)	1475 (11.9)		ref
Missing	200 (21.6)	701 (15.1)			1265 (10.2)	40 (7.9)		
Dressing								
Independent	647 (69.7)	3319 (71.6)	0.001	1.57 (1.19–2.05)	391 (77.6)	9219 (74.7)	0,856	0.98 (0.75–1.27)
Dependent	80 (8.6)	618 (13.3)		ref	75 (14.9)	1854 (15.0)		ref
Missing	201 (21.7)	699 (15.1)			38 (7.5)	1276 (10.3)		

## Discussion

This case-control observational study based on the Swedish stroke registry, Riksstroke, revealed that ESD provided significant benefits to patients with stroke treated in modern stroke care units. Those that received ESD experienced more satisfaction with rehabilitation after discharge, less need for assistance with ADL, and less dysthymia/depression, compared to patients that did not receive ESD.

The outcomes of this study were based on the responses to PROMs. PROMs evaluate a person’s feeling: it includes symptom reporting, satisfaction with care and treatments, self-rated health, and health-related quality of life [13]. In a large registry with national coverage (Riksstroke), it is important to choose simple questions that cannot be misunderstood. Rikstroke has developed simplified PROMs for dysthymia/depression, fatigue, pain, general health status and ADL (mobility, toileting and dressing). These PROMs have been validated against more established measurements (http://www.riks-stroke.org). The validation showed good agreement with established measurements and accurate reliability for the variables chosen in this study (http://www.riks-stroke.org, [14]).

In the current study, it was possible to identify and exclude those who did not receive ESD in the intervention group, but it was not technically possible in the control group. About 2% of the participants in the control group had received ESD, according to Rikstroke data. These participants were distributed throughout the control hospitals, and the majority of hospitals reported only a few participants/year. It is possible that these registrations are incorrect and we assumed that these patients had not received rehabilitation according to an established model for ESD and that including these patients would not affect our analysis.

Patient satisfaction regarding ESD has previously been evaluated, but those studies reported conflicting results. Some studies showed improved satisfaction with ESD [1, 15] and others found insufficient evidence [8]. Patient satisfaction may be affected by various factors, particularly homecoming experiences and patient expectations. Some studies have investigated patient experiences in homecoming and home rehabilitation in the context of ESD [16–18]. Those studies indicated that patients and their families were positive to coming home, and they expressed great satisfaction about receiving rehabilitation within the home environment. The patients felt more capable of undertaking ADL in the home environment, and they were looking forward to re-establishing meaningful participation in their chosen life roles [16]. Previous research has also reported that domiciliary therapy allowed patients and their families to become more involved in decision-making, to provide more insight, and to offer motivation in rehabilitation functions [19].

Another aspect of satisfaction with rehabilitation is the patient’s expectations of coming home with ESD after stroke. A recent study [20] on the expectations of coming home with very early supported discharge and home rehabilitation after stroke found that the participants had mixed expectations. Patients were longing to come home, but also described insecurity and fear. Despite these mixed expectations, the participants were highly confident that the ESD team would support them in achieving independence [20]. Presumably, patient satisfaction was closely linked to their participation in rehabilitation. Thus, it was important that patient expectations were consistent with the intervention received.

A strength of this study was the large control group, which was drawn from many different hospitals covered in Riksstroke. Another strength was the use of two registries, which provided information about both patient characteristics, education level, and country of birth. We adjusted for variables that were measured in Riksstroke and differed in the baseline comparison, but as in all observational studies, there is a possibility of unmeasured confounding. Since we expected the outcome variables to be correlated, we chose not to adjust the confidence level for multiple testing. Using Bonferroni correction, which is a conservative method, we would still report significant results for outcome with *p*-values < 0.005 (all but toileting and dressing).

A limitation in Rikstroke in general is a limited response rate of NIHSS at baseline thus excluding detailed description of stroke severity. In a previous observational implementation study describing the ESD at Umeå Stroke Center, a modified version of the NIHSS at admission that included level of consciousness, arm and leg paresis and language showed a mean value of 2 ± 2.2 (SD). In this study we aimed to model positive outcome. In general, there were more missing responses in the ESD group. In the sensitivity analysis (Table 3) we included missing responses in the negative category (which corresponds to a worst-case scenario). This analysis showed similary results as the main analysis in Table 2. The lack of response may be due to different causes. In this study, about 30% of the participants in the ESD group reported that they had no need of rehabilitation after discharge. However, according to information in the Riksstroke Registry, all individuals in the ESD group received rehabilitation efforts. This highlight the difficulty of collecting PROM responses at 3 months after a stroke onset. After a stroke, it is not unusual for an individual to have problems, initially, with memory, concentration, and fatigue, and all these symptoms can affect the reliability of the PROM response. The lack of response can also be due to a wish to continue life and put the stroke incidence behind. In addition, it is important to consider a recall bias: that is, by the time a patient responds to the 3-months Riksstroke follow-up survey, it may be difficult to recall their experience with rehabilitation after discharge. Difficulties to recall their experience with rehabilitation after a long time and the fact that this is a fragile group, where the health can change is the reason for not including 12-months follow up in this study.

We found no differences between the ESD vs no ESD groups in pain or fatigue. This finding may be explained by the fact that a treatment recommendation was established for depression, but no consensus was established regarding the treatment of pain and/or fatigue.

Our results concerning ADL dependency were consistent with previously reported results. Patients that received ESD required less ADL assistance than patients that received modern conventional care without ESD [1, 8, 21].

## Conclusions

This case-control observational study, based on registry data of PROMs at 3-months follow up showed that patients that received ESD after stroke were more satisfied with rehabilitation after discharge and experienced less depression than patients that received other health care and/or rehabilitation care. In this study, all patients received modern stroke unit care with short hospital stays, access to hyper-acute therapies, and early carotid interventions. Therefore, our results substantiate the hypothesis that the previously shown benefits of ESD and home rehabilitation were also observed among patients treated in modern stroke care.

## Abbreviations

ADL: Activities in daily living
CI: Confidence Interval
ESD: Early supported discharge
LISA: Longitudinal Integration Database for Health Insurance and Labor Market Studies
NIHSS: National Institutes of Health Stroke scale
OR: Odds Ratio
PROM: Patient-reported outcome measurement
REF: Reference
RLS85: Reactive Level Scale
SD: Standard Deviation
SU: Stroke unit
